# Patient Satisfaction Following Orthodontic Treatment: A Systematic Review

**DOI:** 10.7759/cureus.65339

**Published:** 2024-07-25

**Authors:** Abdulmalek M.H. Almasri, Mohammad Y. Hajeer, Mowaffak A. Ajaj, Alaa Oudah Ali Almusawi, Samer T. Jaber, Ahmad Salim Zakaria, Mohammad Khursheed Alam

**Affiliations:** 1 Department of Orthodontics, Faculty of Dentistry, University of Damascus, Damascus, SYR; 2 Department of Orthodontics, Faculty of Dentistry, University of Al-Knooz, Basrah, IRQ; 3 Department of Orthodontics, Faculty of Dentistry, Al-Wataniya Private University, Hama, SYR; 4 Department of Orthodontics, School of Dental Sciences, University Sains Malaysia, Kelantan, MYS; 5 Department of Preventive Dental Science, College of Dentistry, Jouf University, Sakaka, SAU

**Keywords:** patients’ satisfaction, removable appliances, fixed appliances, patient-centered care, patient-reported outcome measures, orthodontic treatment

## Abstract

Patient-reported outcome measures (PROMs) have become increasingly important in orthodontic treatment as they reflect patients' perceptions of treatment outcomes. Understanding patient satisfaction with orthodontic treatment is crucial for improving healthcare delivery and patient-centered care. This systematic review aimed to critically appraise the evidence regarding patient satisfaction after orthodontic treatment, exploring the effects of different treatment types, patient demographics, and other factors on satisfaction levels. Eight electronic bibliographic databases were searched without publication time or language restrictions, including PubMed®, Scopus®, the Cochrane Central Register of Controlled Trials, Web of Science™, Embase®, Google™ Scholar, Trip, and OpenGrey. A manual search was conducted on the references in the included papers. Eligibility criteria were established based on the Population, Intervention, Comparison, Outcomes, and Study (PICOS) framework. Studies were included if they reported patient satisfaction levels following orthodontic treatment using standardized questionnaires. Two reviewers independently collected and analyzed the data. The risk of bias was assessed using Cochrane’s risk of bias tool (RoB2) for randomized clinical trials, and the methodologic quality for cohort and cross-sectional studies was assessed using the modified version of the Newcastle-Ottawa scale. Fourteen studies employed various questionnaires and timings to gauge post-orthodontic treatment satisfaction. Patient satisfaction levels were generally high, with most studies reporting satisfaction rates above 91%. Fixed orthodontic appliances were associated with higher satisfaction levels compared to removable appliances. While age and gender did not significantly influence satisfaction, the quality of care and doctor-patient relationships were crucial factors in patient satisfaction. This systematic review proves that patient satisfaction with orthodontic treatment is generally high, with fixed appliances and positive doctor-patient relationships contributing to higher satisfaction levels. However, the quality of the evidence was moderate to low, highlighting the need for further high-quality clinical studies in this area.

## Introduction and background

Recently, interest in patient-reported outcome measures (PROMs) during healthcare provision has grown significantly due to their relationship with treatment success, patient cooperation, and patient satisfaction with the results achieved [1]. Therefore, patients' perceptions of their treatment planning and expectations have become increasingly crucial in justifying health services delivery and ensuring overall healthcare quality [2].

Patient satisfaction is one of the most important things orthodontists seek to achieve at the end of orthodontic treatment. The evaluation of treatment outcomes varies between patients, their families, and the orthodontist because patients' assessments are based on subjective rather than technical aspects and are therefore subject to the influence of various factors, such as personality type, socioeconomic status, age, and gender [3]. Therefore, no proof that patient satisfaction and professional evaluation are directly correlated exists [4].

Different approaches have been used to assess patient satisfaction after orthodontic treatment. These methods have been based primarily on patients' subjective opinions, not professional evaluations [5,6]. Questionnaires are generally used to detect patient satisfaction, and these are filled out during follow-up clinical sessions [7-9] or mailed to patients' addresses following treatment [10]. In addition, structured interviews are sometimes used to obtain patients' responses [11] or sometimes by phone [12]. On some occasions, the researcher may depend on both modalities of outcome assessment (i.e., interviews and questionnaires) [13,14].

Investigations have shown a wide range of patient satisfaction levels after orthodontic treatment [15]. A study by Al-Omiri and Abu Alhaija [16] evaluated patient satisfaction after fixed appliances orthodontic treatment using the dental impact on daily living (DIDL) questionnaire, where half of the patients were treated with extraction and the other half were treated with non-extraction. Total satisfaction scores showed that 4% of the treated patients were dissatisfied with their teeth; all were treated non-extraction, 62% were relatively satisfied, and 34% were totally satisfied with their teeth. On the other hand, Anderson et al. [5] stated that 2.8% of patients reported severe dissatisfaction with their orthodontic care, 22.6% had moderate satisfaction, and 74.6% reported high treatment satisfaction. Another study by Maia et al. [17] evaluated Angle Class I and II patient satisfaction using the DIDL questionnaire to collect patients' responses and showed that 77.5% of the sample reported being satisfied with their dentition, while 22% were relatively satisfied, and 0.5% reported dissatisfaction.

Two systematic reviews have been published evaluating factors affecting patient satisfaction after orthodontic treatment [18,19]. Still, no systematic review has been published with the main objective of critically and systematically appraising the available evidence regarding satisfaction levels after orthodontic treatment. Therefore, the current systematic review aimed to synthesize the evidence regarding satisfaction levels following orthodontic treatment. The central research question addressed in this report was ‘What is the level of patient satisfaction after orthodontic treatment?

## Review

Scoping search

Before drafting the final systematic review procedure, a PubMed scoping search was conducted to confirm previous systematic reviews' existence and identify potentially suitable publications. This systematic review followed the Preferred Reporting Items for Systematic Reviews and Meta-Analyses (PRISMA) guidelines [20].

Review inclusion and exclusion criteria

The Participants, Intervention, Comparison, Outcomes, and Study Design (PICOS) framework was utilized in the search strategy. The participants should be healthy patients of both genders, at any age and from any ethnicity, who underwent orthodontic treatment with fixed or removable orthodontic appliances. The intervention group should include any conventional orthodontic treatment without any acceleration method of orthodontic tooth movement. In comparative studies, the comparison group should include patients treated with another orthodontic fixed appliance technique that is different from those in the interventional group regarding the type of brackets, type of ligation, or type of prescription. However, if the comparison group included untreated patients, this study would be included in the review. The comparative group should not be subjected to an orthodontic acceleration technique in all different scenarios. The outcome measures under assessment should be patient satisfaction after orthodontic treatment measured by a visual analog scale, numerical rating scale, verbal rating scale, the dental impact of daily living scale, or any other validated patient satisfaction questionnaire. Concerning the included study designs in this review, randomized or non-randomized controlled trials (RCTs/ CCTs), cohort studies, and cross-sectional studies were accepted without publication time or language restrictions.

Information sources

An electronic literature search was performed using PubMed®, Scopus®, the Cochrane Central Register of Controlled Trials, Web of Science™ Embase®, Google™ Scholar, Trip, and OpenGrey. A manual search was conducted on the references in the included papers to find any other pertinent research that might have been overlooked during the computerized searches. ClinicalTrials.gov and the World Health Organization’s International Clinical Trials Registry Platform were also electronically reviewed to identify ongoing, completed, yet published clinical trials.

Search strategy and study selection

Appendix 1 contains a list of the keywords utilized in the search strategy. Details of the electronic search strategy are mentioned in Appendix 2. Two phases were involved in determining the selected articles' eligibility. In the first stage, the titles and abstracts about satisfaction with orthodontic treatment found by all electronic databases were examined separately by two reviewers (AMM and MYH). In the second stage, the reviewers evaluated the full-text articles to determine their final eligibility. Disagreements between them were solved by a third review author (MAA), who reached a decision when necessary.

Data collection process

Two reviewers (AMM and MYH) extracted data from the included studies and arranged them into tables and when there was a disagreement, the third author (STJ) was tasked with resolving it until a consensus was achieved. The following details are included in the tables: general information (author names, study setting, and publication year), methods (study design, questionnaire type), participants (sample size, age, type of malocclusion), treatment type, and satisfaction evaluation timing.

Assessing the risk of bias of the included studies

Initially, the risk of bias for the included articles was determined by the two reviewers (AMM and MYH) separately using Cochrane’s risk of bias tool for randomized trials (RoB2) [21] and the modified version of the Newcastle-Ottawa scale for non-randomized trials [22]. After that, the two reviewers' assessments were compared; in case of disagreement, a third reviewer (ASZ) was asked to help reach a decision. For RCTs, the following five domains were judged as unclear, low, or high risk of bias: randomization process, deviations from intended interventions, missing outcome data, measurement of the outcome, and selection of the reported result. Then, each study’s overall risk of bias was determined based on the following criteria: a high risk of bias occurs when one or more domains are evaluated as having a high risk of bias; a moderate risk of bias occurs when one or more domains are evaluated as having an unclear risk of bias. A low risk of bias occurs once all domains have a low risk.

The modified Newcastle-Ottawa scale was intended for cross-sectional, cohort, and case-control research. This tool uses eight domains, further divided into three primary categories, to evaluate the studies: patient selection, comparison of study groups, and outcome assessment. A rating system was employed to assess study quality. High-quality studies with minimal bias could receive up to 9 stars, while those with 8, 7, or 6 stars were considered moderate quality. Studies of lower quality received five stars or fewer.

Quality of the evidence

The two reviewers (AMM and MYH) independently evaluated the quality of the evidence for each outcome. Subsequently, the judgments of both reviewers were compared. In case of disagreement and a conversation was not resolved, a third reviewer (MKA) was consulted to help reach a decision.

Results

Literature Search Flow and the Retrieved Studies

**Figure 1:**
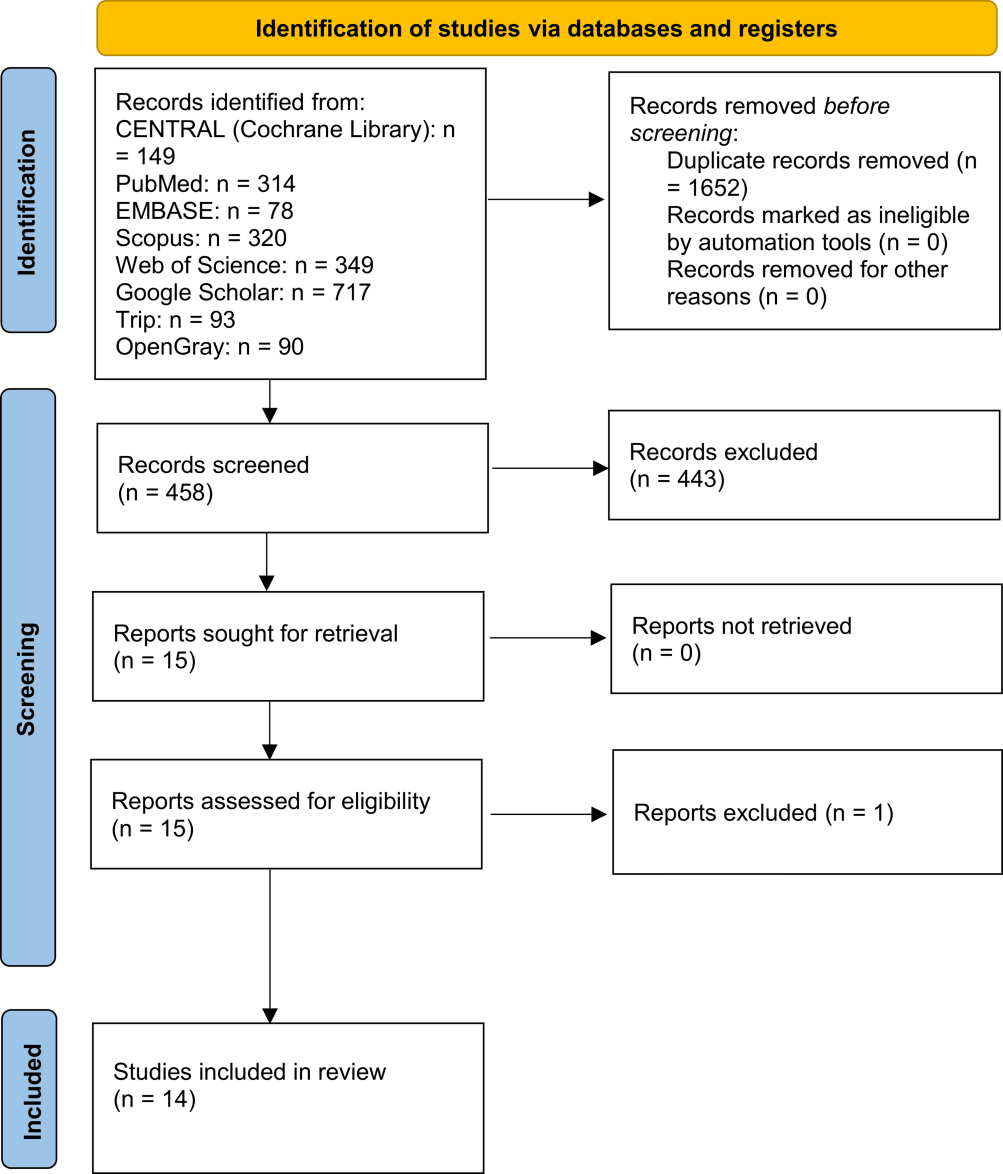
The Preferred Reporting Items for Systematic Reviews and Meta-Analyses (PRISMA) flow diagram of study identification, screening, and inclusion

An electronic search across databases and reference lists produced 2,110 references. After eliminating duplicates, 458 citations underwent a thorough examination. Subsequently, 443 documents were excluded based on title and abstract screening, leaving 15 full-text records for eligibility assessment. Ultimately, the systematic review included 14 studies [5,16,17,23-33]. One was excluded due to the orientation of the satisfaction questionnaire toward the provided treatment procedures rather than the orthodontic outcome. The PRISMA flow chart for the processes for inclusion and selection is presented in Figure 1.

Characteristics of the Included Studies

The features of the included studies are provided in Table 1. Out of these trials, one was an RCT [26], two were cohort studies [27,30], and the other 11 studies had a cross-sectional design [5,16,17,23-25,28,29,31-33]. They were all in English. These studies were carried out across 13 countries, including the UK [25], Brazil [17], Netherlands [24,29], the USA [5], China [31], Turkey [33], Saudi Arabia [23], Jordan [16], Syria [30], Sweden [27], Canada [28], Belgium [26], and Norway [32].

**Table 1:** Characteristics of the included studies VAS: visual analog scale, PSQ: patient satisfaction questionnaire, DIDL: dental index of daily living, PSPSQ: post-surgical patient satisfaction questionnaire, RCT: randomized controlled trial, OPTIQ: orthodontic patient treatment impact questionnaire, NRS: numeric rating scale

Author, Year, and Country	Study design	Patients (M/F) and Mean age (years)	Type of malocclusion	Type of treatment	Timing of satisfaction assessment	Satisfaction assessment tool
Bos et al., 2005, Netherland [24]	Cross-sectional	Patients (M/F):100 (44/56); Mean age: 15.81±1.81	Different types of malocclusion	Fixed appliance treatment	Three years after the end of active orthodontic treatment	Non-validated questionnaire with 20 items graded on 5-point Likert scale
Al-Omiri and Abu Alhaija, 2006, Jordan [16]	Cross-sectional	Patients (M/F):50 (30/20); Mean age: 20.7±4.2; Age range: 13-28	Different types of malocclusion	Fixed appliance treatment (50% with extraction and 50% without extraction)	After the end of the retention phase that prolonged 6-12 months	Validated DIDL questionnaire
Uslu and Akcam, 2006, Turkey [33]	Cross-sectional	Patients (M/F):40 (13/27); Mean age: 13.4±4.1	Class III malocclusion	Functional therapy followed by fixed appliance	At least five years after retention	Non-validated questionnaire with 13 items graded on 4-point Likert scale
Anderson et al., 2009, USA [5]	Cross-sectional	Patients (M/F): 147 (41/106); Mean age: 11.61±1.92	Different types of malocclusion	Different types of orthodontic treatment	Maximum of 3.5 years post-orthodontics	A modified PSPSQ questionnaire
Maia et al., 2010, Brazil [17]	Cross-sectional	Patients (M/F):209 (70/139); Mean age: 14.3	Class I or II malocclusion	Fixed appliance treatment with or without functional therapy 14.4% with extraction	5-25 year post-orthodontics	Validated DIDL questionnaire
Keles and Bos, 2013, Netherlands [29]	Cross-sectional	Patients (M/F):115 (40/75); Mean age: 17.23±3.76	Different types of malocclusion	Different types of orthodontic treatment	Three year post-orthodontics	Validated questionnaire with 15 items graded on a 5-point Likert scale
Feldmann, 2014, Sweden [27]	Cohort	Patients (M/F):120(60/60); Mean age: 14.3±1.73	Class II malocclusion or bimaxillary protrusion	Fixed appliances with two or four premolar extraction	On the first visit of retention	Questionnaire graded on VAS
Li et al., 2016, China [31]	Cross-sectional	Patients (M/F):120 (60/60); Mean age: 13.3 ± 1.73	Class I malocclusion with mild to moderate crowding	Non-extraction fixed appliance treatment	On the first visit of retention	Questionnaire graded on VAS
Aljughaiman et al., 2018, Saudi Arabia [23]	Cross-sectional	Patients (M/F):229 (79/150); Mean age: 22.69±6.34	Different types of malocclusion	Different types of orthodontic treatment	At least one year after the end of orthodontic treatment	Validated Likert-scale questionnaire
Flores-Mir et al., 2018, Canada [28]	Cross-sectional with 2 groups	Patients (M/F):122 (33/89); age range: 18-25	Different types of malocclusion	81 treated with Invisalign clear aligners; 41 treated with fixed appliances	In debonding appointment	Validated PSQ
Charavet et al. 2019, Belgium [26]	RCT	Patients (M/F):24 (9/15); Control group: 12; Test: 12; Mean age: 27.9 ± 7.6	Mild to moderate crowding	Control: fixed appliance; Test: fixed appliance with piezocision	Immediately after treatment completion	Four-item VAS questionnaire
Bradley et al., 2020, UK [25]	Cross-sectional	Patients (M/F):203 (70/133) age range: 12 years and older	Different types of malocclusion	Different types of orthodontic treatment	In the retention stage at variable times.	Validated OPTIQ
Salvesen et al., 2022, Norway [32]	Cross-sectional	Patients (M/F):211 (96/115) age: younger than 18 years	Different types of malocclusion	Fixed appliances treatment	Three to ten years after the end of orthodontic treatment	Ten-item dichotomous scale in a validated questionnaire
Kusaibati et al., 2023, Syria [30]	Cohort	Patients (M/F):28 (10/18) Mean age: 20.68 ± 1.91	Class I malocclusion with moderate crowding	Non-extraction fixed appliance treatment	At the debonding appointment	Five-item NRS in a validated questionnaire

One thousand seven hundred and eighteen participants were included in these 14 studies (1,063 females and 655 males). All these studies involved participants of both genders, and no studies focused solely on one gender. The findings exhibited large variations in sample sizes (ranging from 24 to 229 patients), ages (range: 11- 51 years old), and time passed since the removal of the orthodontic appliances; some surveys were completed at the debonding appointment [28,30], and others in the retention stage [16,25-27,31], and others years after orthodontic treatment [5,17,23,24,29,32,33]. All of the studies used questionnaires as assessment tools.

Patient satisfaction was evaluated with several types of malocclusions. Mild-to-moderate malocclusion was assessed by two studies [26,31], whereas one study included moderate malocclusion [30]. Children with Class III malocclusion were evaluated in one study [33], whereas patients with Class II malocclusion or bimaxillary protrusion were studied in one paper [27]. One study included patients with class I or II malocclusions [17], while the other included other malocclusions [5,16,23-25,28,29,32].

In seven studies, only fixed orthodontic appliances were used in patients’ treatment [16,24,26,27,30-32]. One study used functional treatment followed by a fixed appliance [33]. In the study of Maia et al., fixed appliances were used with or without functional treatment [17], whereas in the study of Flores-Mir et al., clear aligners or fixed appliances were used [28]. The other four studies used other types of orthodontic appliances [5,23,25,29].

Risk of Bias and Quality of the Included Studies

**Figure 2:**
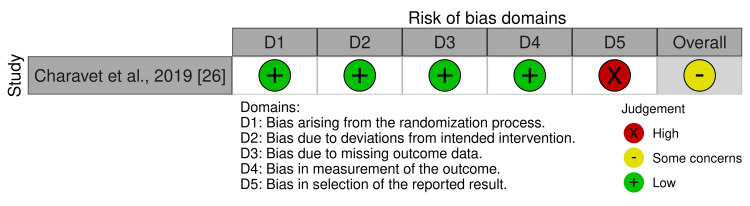
Risk of bias of the included randomized controlled trial

As shown in Figure 2, the only included RCT was classified as having some concern of bias due to selective reporting. Specifically, not all outcomes mentioned in the registered protocol have been reported in the study by Charavet et al. [26]. Appendix 3 provides more details about the risk of bias evaluation of the included RCT.

The methodological quality scores for the other 13 cohort and cross-sectional studies were derived from the Newcastle-Ottawa scale, as shown in Table 2. Eight studies were considered moderate quality, and five were considered low quality. None of the studies was assessed to be of high quality since the maximum of nine stars was not reached in any study. Regarding selecting the study groups, six studies received four stars, and seven scored three or less. The sample size and power calculation were the primary methodologic limitations that indicated the possible risk of bias. Less than half of the research found that the non-respondent selection for cross-sectional studies was insufficient, and most studies could not justify their sample sizes. Samples represented corresponding populations in only six studies [23,25,27,28,31,32]. Concerning the ascertainment of the outcome of interest, 1 star was scored for seven studies, two stars scored for four studies, and three stars scored for the other two studies.

**Table 2:** Quality assessment for non-randomized studies using the modified Newcastle-Ottawa scale Studies considered high quality and at low risk of bias can receive a maximum of 9 stars, whereas articles achieving 8, 7, or 6 stars have moderate quality, and articles with 5 stars or fewer indicate low quality.

Study	Selection (****)				Comparability (**)	Outcome (***)		Total score
	Representativeness of the sample	Sample size	Non-respondents	Ascertainment of exposure	The subjects in different outcome groups are comparable, based on the study design or analysis; confounding factors are controlled.	Assessment of the outcome	Statistical test	
Bos et al., 2005 [24]	*	*	*	*	*	*	-	6
Al-Omiri and Abu Alhaija, 2006 [16]	-	*	*	**	*	*	*	7
Uslu and Akcam, 2006 [33]	-	-	-	*	*	*	-	3
Anderson et al., 2009 [5]	*	*	*	*	-	*	*	6
Maia et al., 2010 [17]	*	*	-	*	*	*		5
Keles and Bos, 2013 [29]	*	*	-	*	*	-	*	5
Feldmann, 2014 [27]	*	*		**	*	**	*	8
Li et al., 2016 [31]	*	*	*	*	*	-	*	6
Aljughaiman et al. 2018 [23]	-	*	*	*	*	*	*	6
Floris-Mir et al., 2018 [28]	-	*	*	*	-	-	*	4
Bradley et al., 2020 [25]	*	-	*	*	*	*	*	6
Salvesen et al., 2022 [32]	*	-	*	*	*	-	*	5
Kusaibati et al., 2023 [30]	*	*	*	*	*	**	*	8

Main Findings

Effects of orthodontic treatment on patient satisfaction: All the included studies assessed the influence of orthodontic treatment on patient satisfaction in this systematic review. All the studies reported high patient satisfaction after the orthodontic treatment. Most of the articles used different questionnaires at different assessment times to assess satisfaction with orthodontic treatment, and the extracted data (sample size, male-female ratio) were heterogeneous; therefore, it was impossible to perform a meta-analysis.

Effect of type of orthodontic treatment on patient satisfaction: The impact of only fixed orthodontic treatment on patient satisfaction was investigated in seven studies [16,17,26,27,30-32]. Despite variations in satisfaction assessment timing, high levels of satisfaction were achieved in all the studies, ranging from 91% in the study by Li et al. [31], which evaluated patient satisfaction at the first retention visit, to 99.5% in the study by Maia et al. that evaluated patient satisfaction after 5-25 years after orthodontic treatment [17]. Treatment with functional therapy followed by fixed appliances was addressed by Uslu and Akcam [33], and 90% of treated patients were satisfied with the final result. Four studies have examined the effect of extraction treatment on patient satisfaction [16,17,27,32]. No association was observed between patient satisfaction and orthodontic extraction in two studies [17,32].

In contrast, Al-Omiri and Abu Alhaija found that 8% of patients treated with non-extraction were dissatisfied with the final results, while all the patients treated with extraction were satisfied [16]. Moreover, in two studies, all the patients were treated non-extraction. The cohort study by Kusaibati et al., which assessed patient satisfaction at the debonding appointment, reported a remarkable satisfaction rate of 96% [30]. Similarly, Li et al.'s cross-sectional study, which evaluated patient satisfaction during the first retention visit, found a satisfaction rate of 91% [31]. 

Effect of age and gender on patient satisfaction: The relationship between patients’ gender and satisfaction after orthodontic treatment was investigated in eight studies [16,17,24,27,29,30,32,33]. Among these, four studies examined the impact of patients’ ages and gender on patient satisfaction [16,17,29,30]. Regarding patients’ gender, six studies reported no significant correlation between gender and patient satisfaction with orthodontic treatment [16,17,27,29,30,32]. However, Bos et al. found that female patients exhibited higher satisfaction levels in the context of the doctor-patient relationship and the situational aspects of treatment. Specifically, they reported greater contentment with their interactions with healthcare providers and the practical aspects of their treatment [24]. Uslu and Akcam conducted a study on class III malocclusion patients, treating them with functional therapy followed by fixed appliances. They also found that gender significantly affects patient satisfaction, especially concerning general facial appearance. There were statistically significant differences in the rate at which women said they were “very satisfied” or “satisfied” with their general facial appearance in comparison with men (P< 0.001). Whereas 70.4% of females expressed “very satisfied” with their overall facial appearance, only 23.1% of males responded similarly [33]. As for patients’ age, three studies found no significant correlations between age and satisfaction scores [16,17,29]. In contrast, Kusaibati et al. discovered a moderate positive correlation between overall satisfaction scores and age, specifically regarding the final treatment results [30].

Discussion

Two prior systematic reviews have been conducted in this field, each with a distinct focus. The first review delved into the domain of patient satisfaction, specifically examining it concerning long-term stability [18]. The second review embarked on a comprehensive exploration of the various factors associated with patient satisfaction [19]. However, a gap exists in the literature as no systematic review has specifically addressed the levels of satisfaction associated with different types of orthodontic treatment. This makes the current review of paramount importance. It is the first systematic review attempting to synthesize the evidence regarding satisfaction levels following orthodontic treatment. Bridging this gap provides a more comprehensive understanding of patient satisfaction in the context of orthodontic treatment. Many recent studies have been conducted in this field, addressing patient satisfaction following accelerated orthodontics [34-39]. All the trials that utilized any acceleration method were excluded to neutralize any possible effect of the acceleration interventions on patient satisfaction.

Effects of Orthodontic Treatment on Patient Satisfaction

An agreement was observed between the included studies regarding high levels of overall patient satisfaction following orthodontic treatment. This agreement can be explained by its aesthetic and functional improvements and the psychological benefits, such as increased self-confidence and self-esteem [25]. The quality of care and positive interactions with the dental team also contribute to this satisfaction. However, clear communication about treatment outcomes is crucial to meet patients’ expectations and ensure their satisfaction. Despite the generally high satisfaction levels, it’s crucial to remember that every patient experience is unique, and the level of satisfaction may differ between individuals [5].

The Effect of Type of Orthodontic Treatment on Patient Satisfaction

Seven studies addressed patient satisfaction following fixed orthodontic treatment alone [16,17,26,27,30-32]; despite differences in patient ages, types of malocclusion, and the timing of questionnaire administration, all studies reported a high degree of satisfaction. This outcome can be attributed to the fact that fixed orthodontic treatment typically enables rapid correction of teeth alignment, which can positively influence patient satisfaction, as the beautiful and well-aligned smile may boost patients’ confidence and improve their appearance, which can, in turn, improve their overall satisfaction.

Four studies addressed the influence of extraction treatment on patient satisfaction [16,17,27,32]. Maia et al. and Salvesen et al. found no correlation between orthodontic extraction and patient satisfaction [17,32]. Conversely, the research conducted by Al-Omiri and Abu Alhaija revealed that 8% of patients who underwent non-extraction treatment expressed dissatisfaction with the outcome [16]. This discrepancy can be attributed to the difference in the patient demographics across the studies. The participants in the AL-Omiri and Abu Alhaija study were adults, whereas the subjects in the studies by Maia et al. and Salvesen et al. were children and adolescents; perhaps it is easier to achieve satisfaction from younger patients, while older patients are more aware that in cases where the decision to extract is borderline, the extraction treatment could potentially lead to an improved facial profile and better teeth alignment, thereby enhancing patient satisfaction.

The Effect of Gender on Patient Satisfaction

Gender was significantly related to satisfaction in two studies [24,33]. In contrast, it was not linked with patient satisfaction in six studies [16,17,27,29,30,32]. Bos et al. found that female patients exhibited higher satisfaction levels in the context of the doctor-patient relationship and the situational aspects of treatment [24]. This may be because their increased familiarity with dental services may lead to more realistic expectations, which are more likely to be fulfilled. Compared to men, women who undergo orthodontic treatment tend to perceive that they receive relevant information during treatment and experience a positive ambiance in the treatment room.

Uslu and Akcam also found that gender plays a significant role in patient satisfaction, especially concerning general facial appearance. 70.4% of females said they were “very satisfied,” with only 23% of men responding similarly [33]. This may be because the subjects in this study had a Class III malocclusion, and the female patients gave higher satisfaction levels because the Class III profile affects females more negatively than males [40].

Limitations of the Current Systematic Review

One primary review limitation is that all the articles exhibited moderate-to-low-quality methodology. Consequently, the confidence in the obtained findings is somewhat compromised. Another limitation is the heterogeneity across the included studies, particularly regarding patient demographics, malocclusion types, questionnaire variations, and assessment timing. Unfortunately, due to these variations, a meta-analysis could not be performed to estimate the treatment effect precisely.

## Conclusions

Based on limited available evidence with a moderate to low level of quality, patient satisfaction levels with orthodontic treatment were generally high. Patients reported higher satisfaction levels with fixed appliances compared to removable ones. However, there is no statistically significant correlation between age and gender in relation to satisfaction following orthodontic treatment. Overall, satisfaction appears to be linked to positive esthetic outcomes, perceived psychological benefits, and the quality of care provided by the doctor-patient relationship. Conversely, dissatisfaction tends to be associated with longer treatment durations and non-extraction treatment.

## Appendices

**Table 3:** Appendix 1: Keywords used in the search strategy

Components of the search strategy	Relevant keywords
Orthodontics	Orthodontic Treatment, Orthodontic Therapy, Orthodontics, Tooth Movement, Orthodontic Tooth Movement, Tooth Displacement.
Satisfaction	Satisfaction, Patient Preference, Dental Impact of Daily Living, DIDL, Quality of Life, PROMs, Patient-Reported Outcome Measures, Satisfaction Likert Scale.
Intervention	Fixed Appliance, Removable Appliance.

**Table 4:** Appendix 2: Electronic search strategy

Database	Search strategy
CENTRAL	#1 orthodontic OR tooth movement OR orthodontic tooth movement OR tooth displacement OR orthodontic treatment OR orthodontic therapy OR fixed appliance OR removable appliance. #2 satisfaction OR patient satisfaction OR patient preference OR patient-reported outcome measures OR PROMs OR dental impact of daily living OR DIDL OR quality of life OR satisfaction Likert scale. #3 #1 OR #2 #4 #1 AND #2
EMBASE	#1 orthodontic OR tooth movement OR orthodontic tooth movement OR tooth displacement OR orthodontic treatment OR orthodontic therapy OR fixed appliance OR removable appliance. #2 satisfaction OR patient satisfaction OR patient preference OR patient-reported outcome measures OR PROMs OR dental impact of daily living OR DIDL OR quality of life OR satisfaction Likert scale. #3 #1 OR #2 #4 #1 AND #2
PubMed	#1 orthodontic OR tooth movement OR orthodontic tooth movement OR tooth displacement OR orthodontic treatment OR orthodontic therapy OR fixed appliance OR removable appliance. #2 satisfaction OR patient satisfaction OR patient preference OR patient-reported outcome measures OR PROMs OR dental impact of daily living OR DIDL OR quality of life OR satisfaction Likert scale. #3 #1 OR #2 #4 #1 AND #2
Scopus	#1TITLE-ABS-KEY (orthodontic OR Tooth movement OR orthodontic tooth movement OR tooth displacement OR orthodontic treatment OR orthodontic therapy OR fixed appliance OR removable appliance. #2 TITLE-ABS-KEY (satisfaction OR patient satisfaction OR patient preference OR patient-reported outcome measures OR PROMs OR dental impact of daily living OR DIDL OR quality of life OR satisfaction Likert scale). #3 #1 OR #2 #4 #1 AND #2
Web of Science	#1TS= (orthodontic OR Tooth movement OR orthodontic tooth movement OR tooth displacement OR orthodontic treatment OR orthodontic therapy OR fixed appliance OR removable appliance. #2TS= (satisfaction OR patient satisfaction OR patient preference OR patient-reported outcome measures OR PROMs OR dental impact of daily living OR DIDL OR quality of life OR satisfaction Likert scale). #3 #1 OR #2 #4 #1 AND #2
Google Scholar	#1 (orthodontic OR Tooth movement OR orthodontic tooth movement OR tooth displacement OR orthodontic treatment OR orthodontic therapy OR fixed appliance OR removable appliance) AND (satisfaction OR patient satisfaction OR patient preference OR patient-reported outcome measures OR PROMs OR dental impact of daily living OR DIDL OR quality of life OR satisfaction Likert scale).
Trip	(orthodontic OR tooth movement OR orthodontic tooth movement OR tooth displacement OR orthodontic treatment OR orthodontic therapy OR fixed appliance OR removable appliance) AND (satisfaction OR patient satisfaction OR patient preference OR patient-reported outcome measures OR PROMs OR dental impact of daily living OR DIDL OR quality of life OR satisfaction Likert scale).
OpenGrey	#1 orthodontic AND satisfaction

**Table 5:** Appendix 3: Risk of bias judgments of the included randomized controlled trial VAS: Visual analog scale

Study	Randomization process	Deviations from intended interventions	Missing outcome data	Measurement of the outcome	Selection of the reported result	Overall bias
Charavet et al., 2019 [26]	Low risk: Sealed envelopes containing the random allocation of each patient to one or the other group were prepared by an independent team and opened as patients accrued.	Low risk: Blinding of participants and people delivering the intervention cannot be performed. We judge that the outcome is not likely to be influenced by a lack of blinding.	Low risk: No dropouts were reported.	Low risk: Data analysis was blinded from the group assignments... And the investigator provided a comprehensive explanation of the use of the VAS and the way to capture the outcome measure for each patient.	High risk: The protocol for the study was registered in clinical trial.gov study ID: (NCT03406130) and not all outcomes mentioned in the protocol have been reported.	Some concerns
